# The Hantzsch reaction for nitrogen-13 PET: preparation of [^13^N]nifedipine and derivatives†

**DOI:** 10.1039/d1cc00495f

**Published:** 2021-04-08

**Authors:** Julia E. Blower, Michelle T. Ma, Fahad A. I. Al-Salemee, Antony D. Gee

**Affiliations:** King's College London, School of Biomedical Engineering and Imaging Sciences, Department of Imaging Chemistry and Biology, 4th Floor Lambeth Wing, St Thomas’ Hospital London SE1 7EH UK julia.blower@kcl.ac.uk antony.gee@kcl.ac.uk

## Abstract

Nitrogen-13 is an attractive but under-used PET radionuclide for labelling molecules of biological and pharmaceutical interest, complementing other PET radionuclides. Its short half-life (*t*_1/2_ = 9.97 min) imposes synthetic challenges, but we have expanded the hitherto limited pool of ^13^N labelling strategies and tracers by adapting the multicomponent Hantzsch condensation reaction to prepare a library of ^13^N-labelled 1,4-dihydropyridines from [^13^N]ammonia, including the widely-used drug nifedipine. This represents a key advance in ^13^N PET radiochemistry, and will serve to underpin the renewed interest in clinical opportunities offered by short-lived PET tracers.

## Abstract 2

The Hantzsch reaction was adapted to allow the rapid radiosynthesis of ^13^N-labelled 1,4-dihydropyridines, including [^13^N]nifedipine, for preclinical PET imaging.

## Graphical abstract

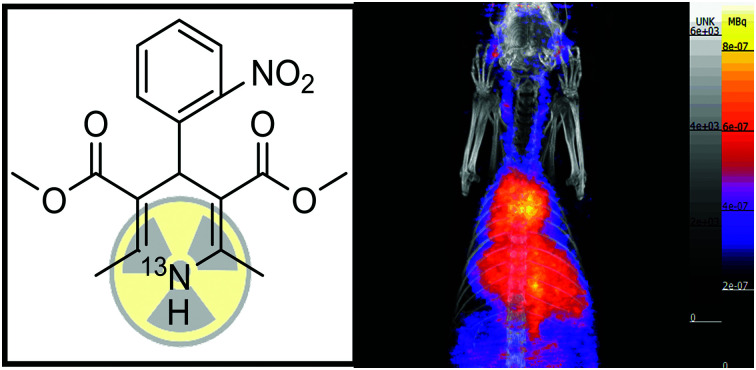

Nitrogen-13 (*t*_1/2_ = 9.97 min, 100% β^+^) is a potentially useful radionuclide for imaging with Positron Emission Tomography (PET). To date nitrogen-13 has been used clinically in the form of [^13^N]NH_3_ for myocardial perfusion imaging.^1^ However, it has been largely over-looked as a viable option for radiolabelling more complex molecules as its very short half-life poses challenges to the development of new synthetic methods.

Despite its limited use, nitrogen-13 has several attractive features. Firstly, the ubiquity of nitrogen in biological and pharmaceutical compounds allows the study of authentic radiolabelled biogenic molecules, which fluorine-18, the most commonly used PET radionuclide, and radiometals very rarely allow. Secondly, whilst many strategies enable biogenic radiolabelling of organic compounds with the PET radionuclide carbon-11, labelling with nitrogen-13 further expands the scope of radiochemical methods available, and labelling the same molecule in alternative positions may give useful metabolic information *in vivo*. Thirdly, unlike fluorine-18 (*t*_1/2_ = 109.7 min) the short half-life of nitrogen-13 allows repeated PET scans on the same individual within a short time period with low radiation dose to the patient. Finally, with the advent of highly sensitive total-body PET scanners that allow for low dose PET imaging, the use of radiopharmaceuticals based on very short-lived radionuclides becomes more feasible.^2^

Single-step multi-component reactions (MCRs) can efficiently generate structurally diverse libraries enabling high-throughput screening.^3^ MCRs have already had some application in the field of radiochemistry: the Ugi, Passerini, Biginelli and Groebke MCRs have been used to access complex ^18^F-labelled structures *via* [^18^F]fluorobenzaldehyde prosthetic groups.^4^ We have labelled peptidic α-aminoacyl amide derivatives and cyclic γ-lactams with nitrogen-13 *via* the Ugi reaction.^5^^11^C-labelled sulfonyl carbamates can be accessed by combining [^11^C]carbon monoxide with sulfonyl azides and alcohols, and the automated synthesis of ^11^C-labelled amino acids *via* the Strecker reaction has been developed.^6,7^

The Hantzsch dihydropyridine (DHP) synthesis is a multicomponent reaction between an aldehyde, two equivalents of a β-keto ester, and an amine (or ammonia). Clinically, 1,4-dihydropyridines including dimethyl 2,6-dimethyl-4-(2-nitrophenyl)-1,4-dihydropyridine-3,5-dicarboxylate (nifedipine), are used as anti-hypertensive and anti-anginal drugs in cardiovascular disease by acting at voltage-gated calcium channels in peripheral blood vessels and the heart;^8,9^ these have been radiolabelled using a range of radionuclides including ^3^H, ^14^C, ^11^C, ^125^I, and ^18^F in order to probe modes of action, pharmacokinetics and metabolism of this class of compounds, and to study the pathophysiology of calcium channels in cardiovascular diseases.^10–21^

To expand the arsenal of ^13^N-radiolabelling tools, we explored the Hantzsch dihydropyridine synthesis as a strategy to access [^13^N]1,4-dihydropyridines ([^13^N]1,4-DHPs) using aqueous [^13^N]ammonia as a synthetic precursor (Fig. 1). These compounds could have clinical utility in imaging the function of calcium channels *in vivo*, providing mechanistic insight into cardiac abnormalities (*e.g.* hypertension, angina, congestive heart failure). The 1,4-DHP scaffold has also exhibited vasodilatory, bronchodilatory, anti-atherosclerotic, hepatoprotective, anti-tumour, anti-diabetic, antimalarial, anti-inflammatory and antibacterial properties.^9,22^ Here we report the first radio-synthesis of a library of ^13^N-labelled 1,4-DHPs using the Hantzsch dihydropyridine synthesis.

**Fig. 1:**
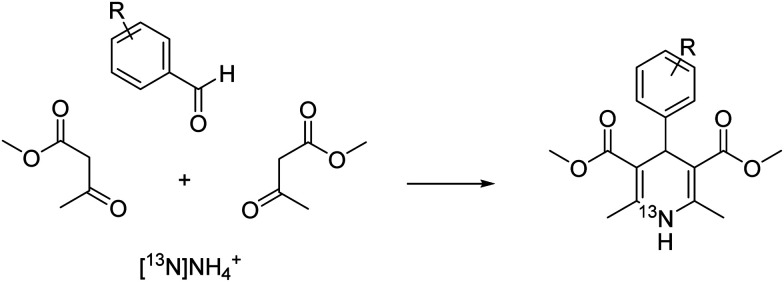
Reaction scheme for the synthesis of ^13^N-labelled 1,4-dihydropyridines.

Prior to exploring the versatility of a Hantzsch MCR radiolabelling strategy, we established optimised radiosynthetic conditions for nifedipine (**1**, Table 1), a calcium channel blocker widely used to treat hypertension and angina. In the first instance, solutions of 2-nitrobenzaldehyde, methylacetoacetate, aqueous [^13^N]NH_3_ (100–150 MBq, cyclotron-produced *via* the ^16^O(p,α)^13^N nuclear reaction with 8 mL H_2_O/ethanol target) and NaOH were combined in DMF and heated in a microwave reactor at 100 °C for 10 min. The crude reaction solution was analysed using reverse-phase radio-HPLC. Encouragingly, the desired product [^13^N]nifedipine, ^13^N-**1**, was present in a radiochemical yield of 20%. This was verified by HPLC co-elution with non-radioactive nifedipine (the non-radioactive “standard” in this case).

**Table 1:** Optimisation for the radiosynthesis of [^13^N]nifedipine (*n* = 1)

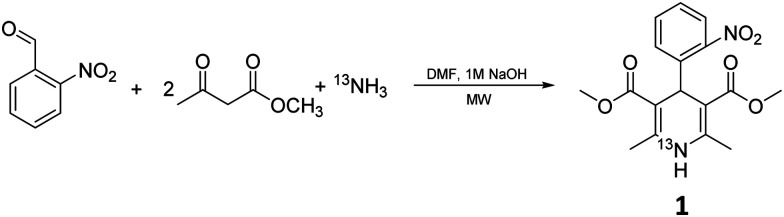			
Entry	*T* (°C)	Time (min)	RCY (%)
1	50	8	64
2	60	8	72
3	80	8	63
4	80	5	79
5	80	3	73
6	100	8	80
7	100	5	85
8	100	3	75
9	120	8	60
10	120	5	65
11	150	8	45
12	150	5	50

Reaction mixtures were also analysed by LC–HRMS to assess whether known intermediates of the Hantzsch MCR were present.^23^ LC–HRMS indicated the presence of methyl-2-(2-nitrobenzylidene)-3-oxobutanoate and dimethyl 2,4-diacetyl-3-(2-nitrophenyl)pentanedioate intermediates (Fig. 2). To probe the reaction pathway further, the reagents 2-nitrobenzaldehyde (5 mmol) and methylacetoacetate (10 mmol) were combined in solution (DMF) and allowed to react at 4 °C. Under these conditions, the rate of reaction of these reagents was sufficiently slow to observe the predicted intermediates formed prior to reaction with ammonia: stereoisomers methyl (*E*)-2-(2-nitrobenzylidene)-3-oxobutanoate (*E*-2a, Fig. 2) and methyl (*Z*)-2-(2-nitrobenzylidene)-3-oxobutanoate (*Z*-2a, Fig. 2), and dimethyl 2,4-diacetyl-3-(2-nitrophenyl)pentanedioate (2b, Fig. 2). Samples of the mixture were analysed periodically using LC–HRMS over the course of five weeks. During this time, signals corresponding to *E*-2a, *Z*-2a and 2b increased (ESI,† Fig. S1). The intermediate species *E*-2a and *Z*-2a were isolated using semi-preparative HPLC and their identity confirmed by ^1^H and ^13^C NMR. With the presence of the intermediates now confirmed in solution, the radiosynthesis of ^13^N-**1** was repeated by combining a “precursor” solution, which contained intermediates *E*-2a, *Z*-2a and 2b, with [^13^N]NH_3_ and NaOH followed by microwave heating. Radio-HPLC analysis showed again the presence of product ^13^N-**1** but now with a significantly improved RCY of 60%. Formation of these intermediates *E*-2a, *Z*-2a and 2b is therefore rate-limiting in the synthesis of ^13^N-**1**. Further optimisation of reaction conditions was carried out on the radiosynthesis of ^13^N-**1** (Table 1). The reaction was observed to be temperature-dependant: an increase in reaction temperature improved yields, achieving a maximum 80% RCY at 100 °C. Heating at temperatures above 100 °C significantly reduced yields to 60% and 50% at 120 °C and 150 °C respectively. Of the short reaction times tested (8, 5 and 3 min to be compatible with the half-life of ^13^N), 5 minutes produced the highest RCY across all temperatures tested. The reaction was observed to be entirely dependent on the presence of sodium hydroxide: its absence resulted in a RCY of 0%. The Hantzsch synthesis typically proceeds under slightly basic conditions, usually provided inherently by the presence of stoichiometric amounts of primary amine or ammonia reagent. Here, with only sub-nanomolar quantities of no-carrier-added [^13^N]ammonia present, supplementing with additional base proved essential for the reaction to proceed, and importantly, under no-carrier-added conditions.

**Fig. 2:**
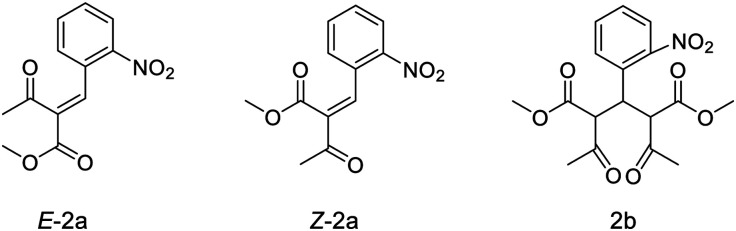
Intermediate species formed in the precursor reaction mixture.

These optimised conditions (100 °C, 5 min) were applied to the synthesis of a library of seven [^13^N]1,4-DHPs containing varying aromatic substituents. In all cases, reaction mixtures containing the respective benzaldehyde derivative and β-keto ester were prepared in DMF, allowed to react, and the presence of the respective *E/Z*-enone and diketone intermediates confirmed *via* LC–HRMS, prior to radiolabelling. Subsequent addition of [^13^N]NH_3_ (100 μL, 100–150 MBq) and sodium hydroxide to these mixtures under the optimised reaction conditions resulted in insertion of ^13^N to furnish the dihydropyridine ring, forming the final ^13^N-labelled 1,4-DHP products **1–7**. All seven 1,4-DHPs were successfully radiolabelled with nitrogen-13 using this adapted Hantzsch synthesis, each confirmed by co-elution with non-radioactive standards, that were either commercially available or prepared using Hantzsch methodology and characterised by ^1^H-NMR, ^13^C-NMR and LC–HRMS (ESI). Excellent radiochemical yields of **1–7** were achieved in the range of 62–95% (Table 2). The labelling of [^13^N]nifedipine (**1**) exhibited a RCY of 80%.

**Table 2:** Analytical HPLC retention times and radiochemical yields of ^13^N-labelled 1,4-dihydropyridines

1,4-DHP	*t*_R_ a (min)	*t*_R_ b ref (min)	Opt. RCY^c^ (%)	Scale-up RCY^d^ (%)
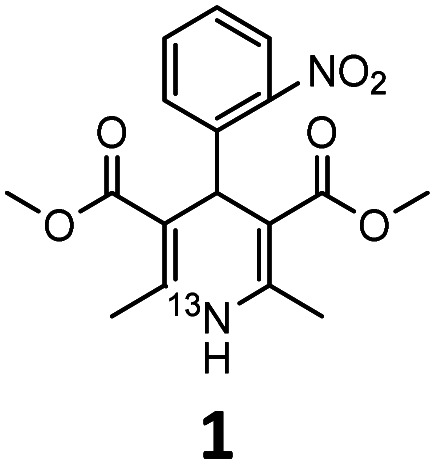	7.85	7.68	80	50
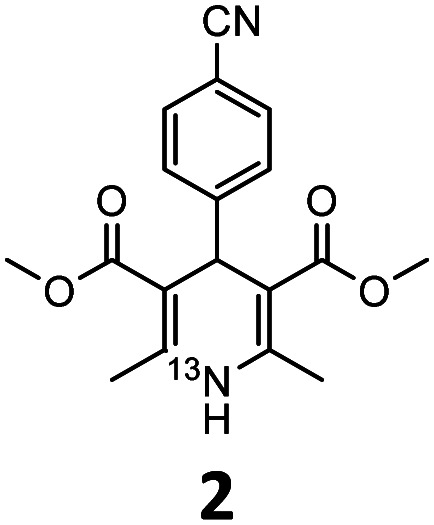	7.98	7.45	95	50
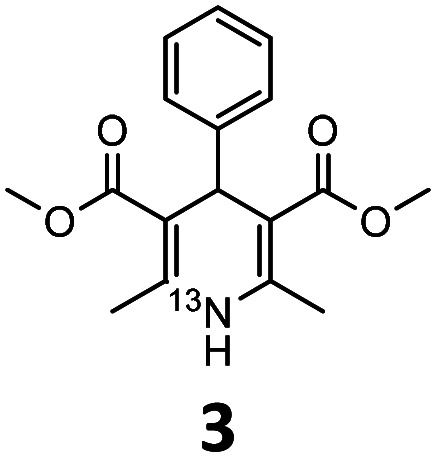	7.90	7.73	90	40
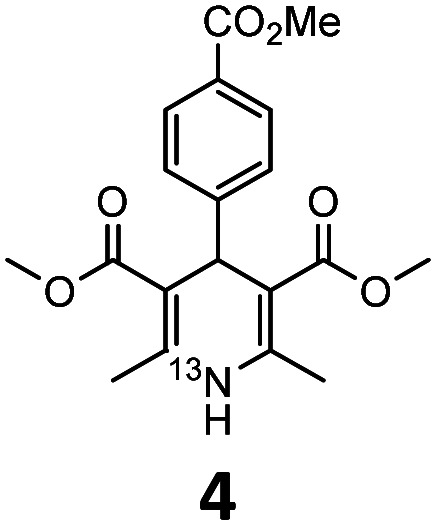	7.83	7.55	95	40
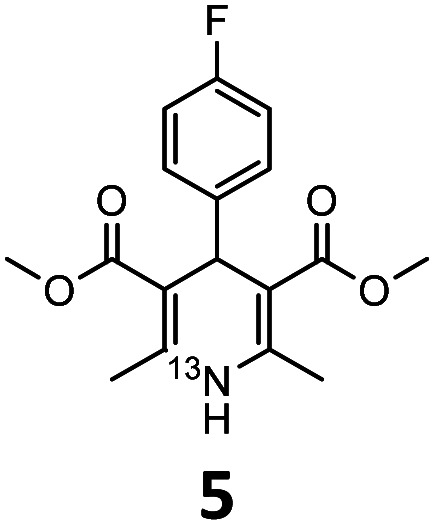	8.22	8.03	91	50
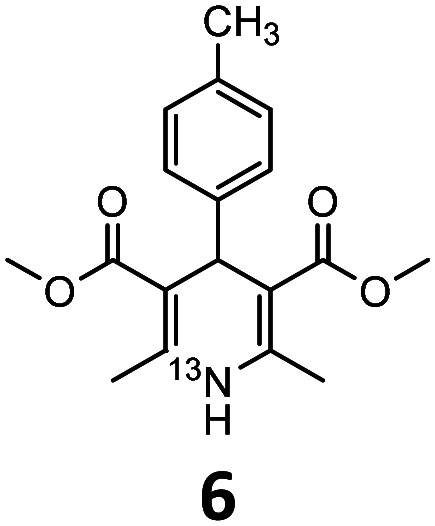	8.50	8.22	73	40
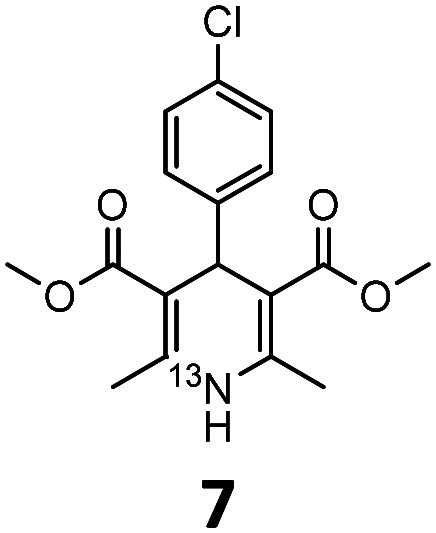	8.82	8.63	62	37

a Retention time of ^13^N-labelled 1,4-DHP on C_18_ analytical HPLC column.

b Retention time of 1,4-DHP reference standard on C_18_ analytical HPLC column.

c Optimum radiochemical yield (before scale-up), decay-corrected.

d Scaled-up radiochemical yield, decay-corrected.

To achieve sufficient quantities of tracer suitable for preclinical PET imaging, the radiosynthesis required further modification. However, simply increasing the aqueous volume of [^13^N]NH_3_ added to the reaction severely reduced the RCY of the final product. To overcome this, [^13^N]NH_4_^+^ was first concentrated further into a smaller volume by solid-phase extraction into a higher concentration of aqueous sodium chloride (1 M).

A modified radiosynthesis was tested: addition of 250 μL aqueous [^13^N]NH_3_ (∼850 MBq) to the solution of reaction intermediates (200 μL) under the revised reaction conditions produced ^13^N-**1** in a reduced RCY of 50%. These conditions were subsequently applied to all reactions to produce ^13^N-labelled products **1–7** with lower RCY (37–50%) (Table 2), but with higher overall amounts of activity, sufficient for preclinical PET imaging. Reversed-phase C_18_ HPLC purification provided **1–7** in 100% radiochemical purity (ESI,† Fig. S3), and for ^13^N-**1**, a molar activity of 174 MBq mmol^−1^ in the final formulation. This radiosynthesis, starting with 850 MBq, took 18 min from start of synthesis to final formulation. However, this “manual” synthesis could be readily adapted to an automated system, enabling use of larger amounts of activity, and shorter overall synthesis and purification times. This would provide [^13^N]nifedipine in higher activities, compatible with clinical use.

To demonstrate the feasibility of [^13^N]1,4-DHPs for PET imaging, [^13^N]nifedipine (**1**, 4 MBq, 800 μL in ethanol/aqueous saline solution) was administered intravenously to a healthy female Wistar rat, and PET/CT acquired over 1 h post-injection (Fig. 3). Accumulation was observed in the blood pool, heart, lung, liver and brain. At 10 min post-injection, radioactivity concentration in the heart was twice that observed in the blood pool, indicating accumulation and retention of [^13^N]nifedipine in myocardial tissue, consistent with the known specific uptake of nifedipine by calcium channels abundant in myocardial tissue.^24^ Uptake in heart, lung and brain also correlated with the known distribution of calcium channels in these organs.^24–26^ Accumulation in the liver was consistent with the known hepatic metabolic pathway of nifedipine *in vivo*: conversion to a nitropyridine analogue *via* oxidative cytochrome P450 3A isozymes.^27,28^ This is followed by further breakdown into polar metabolites most likely excreted *via* the kidneys (not in the field of view).^29^ Broadly, the biodistribution of [^13^N]nifedipine appeared comparable to the behaviour of an ^18^F-labelled nifedipine analogue (in which an [^18^F]CH_2_F group is incorporated in place of one of the CH_3_ groups on the pyridyl ring), with both tracers exhibiting uptake in heart, lung, liver and brain.^30^ Bone uptake was not observed with [^13^N]nifedipine, in contrast with the ^18^F-labelled analogue which showed activity in the skeleton increasing over the course of the imaging procedure, indicative of de-fluorination and release of bone-seeking [^18^F]fluoride. This highlights the advantage of using nitrogen-13 to radiolabel drugs where it is essential to maintain the authentic chemical structure to understand its precise behaviour *in vivo* – even small changes in molecular structure can have large and confounding effects on drug biodistribution and metabolism.

**Fig. 3:**
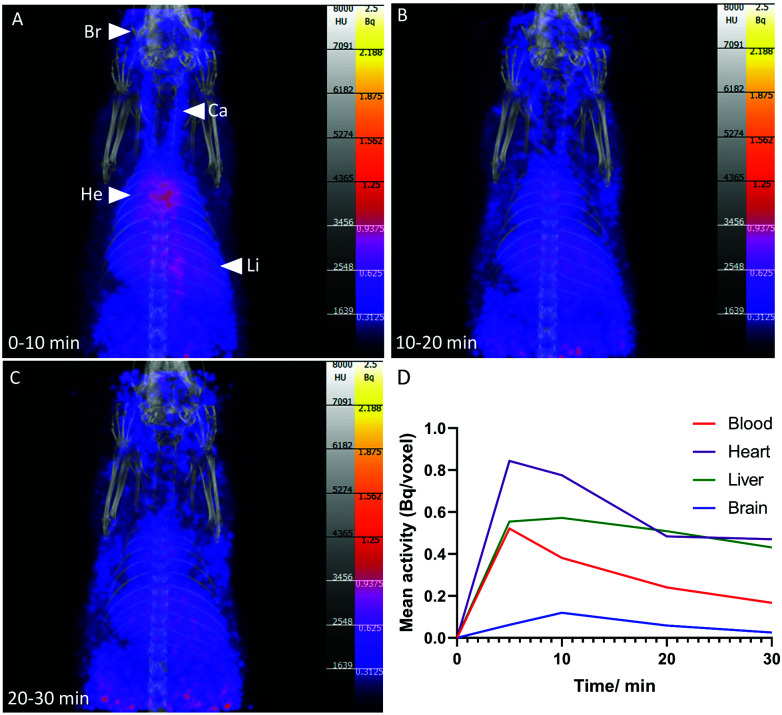
PET/CT maximum intensity projections of [^13^N]nifedipine in healthy rat at (A) 0–10 min; (B) 11–20 min; (C) 21–30 min; Br = brain; Ca = carotid artery; He = heart; Li = liver; (D) mean activity in each organ at 0–30 min obtained from a 4-pixel ROI.

We have shown that the Hantzsch multi-component reaction is suitably versatile for ^13^N-labelling: various substituents can be incorporated on the benzaldehyde ring enabling the rapid development of seven [^13^N]1,4-DHP derivatives from readily available reagents. In these experiments it was convenient to combine the reaction precursors (aldehyde and β-keto ester in DMF) to pre-form enone and diketone intermediates prior to radiolabelling as this led to improvements in radiochemical yield. However, the preparation and isolation of enone and/or diketone intermediates (Fig. 2) could give access to even higher-yielding robust and routine radiochemical syntheses.

The development of new radiosynthetic methods for labelling with biologically and pharmaceutically significant radionuclides like nitrogen-13 and carbon-11 offers opportunities for imaging the whole-body pharmacokinetics of new and existing drugs using authentic radiolabelled biogenic molecules, to inform their early clinical development, which most other radionuclides do not offer. The Hantzsch reaction allows the rapid, no-carrier-added radiosynthesis of 1,4-DHPs from aqueous cyclotron-produced [^13^N]NH_3_, further expanding the arsenal of new ^13^N-labelling methods.

JEB and ADG jointly contributed to the study conception; JEB designed and executed the study; MTM contributed LC-HRMS and NMR acquisition and analysis; FAIA contributed imaging data analysis; JEB wrote the original draft of the manuscript; all authors contributed to manuscript review and editing, and approved the final submission.

The authors would like to thank Dr JB Torres and Mr S Clarke for their assistance with preclinical imaging. This research was supported by the PET Centre at St Thomas’ Hospital and the Department of Health *via* the National Institute for Health Research (NIHR) comprehensive Biomedical Research Centre award to Guy's & St Thomas’ NHS Foundation Trust in partnership with King's College London and King's College Hospital NHS Foundation Trust; a Cancer Research UK Career Establishment Award [C63178/A24959]; the Wellcome Trust funded ‘Multi-User radioanalytical facility for molecular imaging and radionuclide therapy research’ [212885/Z/18/Z]; Wellcome/EPSRC Centre for Medical Engineering at King's College London [WT 203148/Z/16/Z]; the EPSRC programme for next generation molecular imaging and therapy with radionuclides [EP/S019901/1]. The views expressed are those of the author(s) and not necessarily those of the NHS, the NIHR or the DoH.

## Conflicts of interest

There are no conflicts of interest to declare.

## Supplementary Material

CC-057-D1CC00495F-s001
