# Supplementary material for: The Hantzsch reaction for nitrogen-13 PET: preparation of [13N]nifedipine and derivatives
Source: Chem Commun (Camb). 2021 Apr 8;57(40):4962–5. doi: 10.1039/d1cc00495f (PMC8132178; doi:10.1039/d1cc00495f)
Supplement: CC-057-D1CC00495F-s001 [file CC-057-D1CC00495F-s001.pdf]

Electronic Supplementary Material (ESI) for ChemComm.
This journal is © The Royal Society of Chemistry 2021

Electronic Supplementary Information

Chemical Communications

The Hantzsch Reaction for Nitrogen-13 PET: Preparation of [^{13}N]Nifedipine and Derivatives

Julia E. Blower, Michelle T. Ma, Fahad A. I. Al-Salemee and Antony D. Gee

King's College London, School of Biomedical Engineering and Imaging Sciences, Department of Imaging Chemistry and Biology, 4th Floor Lambeth Wing, St Thomas' Hospital, London, SE1 7EH, United Kingdom; Fax: 020 718 85442; Tel: 020 718 88366; Email: julia.blower@kcl.ac.uk; antony.gee@kcl.ac.uk

CONTENTS

SYNTHESIS AND CHARACTERISATION OF 1,4-DHP STANDARDS

PRECURSOR INTERMEDIATE CHARACTERISATION

RADIOLABELLING METHODS

MICROPET/CT IMAGING AND BIODISTRIBUTION

REFERENCES

SYNTHESIS AND CHARACTERISATION OF 1,4-DHP STANDARDS

Chemicals

4-(4-Cl-Ph)-2,6-dimethyl-1,4-2H-pyridine-3,5-dicarboxylic acid dimethyl ester, 2,6-dimethyl-4-phenyl-1,4-dihydro-pyridine-3,5-dicarboxylic acid dimethyl ester, 2,6-dimethyl-4-p-tolyl-1,4-dihydro-pyridine-3,5-dicarboxylic acid dimethyl ester, 4-chlorobenzaldehyde (97%), 4-fluorobenzaldehyde (98%), 4-formylbenzonitrile (95%) and methyl 4-formylbenzoate (99%) were purchased from Aldrich. Dimethyl 2,6-dimethyl-4-(2-nitrophenyl)-1,4-dihydropyridine-3,5-dicarboxylate (nifedipine) was purchased from MP Biomedicals. P-tolualdehyde (98%) and 4-hydroxybenzaldehyde (98%) were purchased from Alfa Aesar. 2-nitrobenzaldehyde (99+%) was purchased from Acros Organics. Benzaldehyde (ReagentPlus®, 99%) was purchased from Sigma-Aldrich.

Instrumentation

Reactions were carried out using a CEM Discover microwave synthesis unit. Radio-HPLC analysis was performed on an Agilent Technologies 1200 Series HPLC with Lablogic β+ detector. Radio-LCMS was performed on Agilent Technologies 6520 Accurate-Mass Q-TOF LC/MS system with Agilent Technologies 1200 Series HPLC connected in series with a UV detector (254 nm) and Lablogic β+ detector. ^{1}H and ^{13}C NMR were acquired using a Brucker DRX 400 MHz. Analytical HPLC and radio-HPLC analysis was performed using an Agilent Eclipse XDB-C_{18} column (5 μM, 4.6 × 150 mm); flow rate 1 mL/min; mobile phase: Solvent A: H_2O + 0.1% TFA, solvent B: MeOH + 0.1% TFA, time:%B, 0:5, 1:5, 10:95, 15:95, 18:5, 23:5. Semi-preparative radioHPLC was carried out using an Agilent Eclipse XDB-C_{18} column (5 μm, 9.4 × 250 mm); flow rate 3 mL/min; mobile phase: solvent A: H_2O + 0.1% TFA; solvent B: MeOH + 0.1% TFA; time (min): %B 0:50, 5:95, 10:95, 15:5, 18:5.

Synthesis of 1,4-DHP standards

For 1,4-dihydropyridine standards not commercially available (dimethyl 4-(4-cyanophenyl)-2,6-dimethyl-1,4-dihydropyridine-3,5-dicarboxylate, dimethyl 4-(4-(methoxycarbonyl)phenyl)-2,6-dimethyl-1,4-dihydropyridine-3,5-dicarboxylate, and dimethyl 4-(4-fluorophenyl)-2,6-dimethyl-1,4-dihydropyridine-3,5-dicarboxylate), substituted benzaldehyde (1 mmol), methylacetoacetate (2 mmol)

and ammonia (28%, excess) were combined in a microwave tube in ethanol (1 mL). The mixture was heated in the microwave synthesis unit at 80 °C for 30 min. The solvent was evaporated under vacuum. The remaining solid was washed successively three times with cold methanol (10 mL × 3) and the solid collected and dried. ^{1}H NMR, ^{13}C NMR and LC-MS analysis was carried out. All compounds are previously known.[1-3]

<u>Analytical characterisation</u>

Dimethyl 4-(4-cyanophenyl)-2,6-dimethyl-1,4-dihydropyridine-3,5-dicarboxylate (2): yellow solid**;** ^{1}H NMR (400 MHz, DMSO-d_6): δ 9.02 (s, 1H, NH), 7.69 (d, *J*=8.3 Hz, 2H, 2CH_{Ar}), 7.31 (d, *J*=8.3 Hz, 2H, 2CH_{Ar}), 4.93 (s, 1H, CH), 3.34 (s, 6H, 2CH_3), 2.27 (s, 6H, 2CH_3); ^{13}C NMR (400 MHz, DMSO-d_6): δ 166.99, 153.03, 146.50, 132.16, 128.04, 118.94, 108.82, 100.58, 50.78, 18.19; LCMS (ESI) $[M+H]^+$ calcd for $C_{18}H_{18}N_2O_4$: 327.13, found: 327.13.

Dimethyl 4-(4-(methoxycarbonyl)phenyl)-2,6-dimethyl-1,4-dihydropyridine-3,5-dicarboxylate (4): pale yellow solid; ^{1}H NMR (400 MHz, DMSO-d_6): δ 8.97 (s, 1H, NH), 7.82 (d, *J*=8.2 Hz, 2H, 2CH_{Ar}), 7.27 (d, *J*=8.3 Hz), 2H, 2CH_{Ar}), 4.94 (s, 1H, CH), 3.80 (s, 3H, CH_3), 3.53 (s, 6H, 2CH_3), 2.26 (s, 6H, 2CH_3); ^{13}C NMR (400 MHz, DMSO-d_6): δ 167.11, 166.11, 153.02, 146.17, 129.12, 127.37, 100.87, 51.93, 50.70, 18.19; LCMS (ESI) $[M+H]^+$ calcd for $C_{19}H_{21}NO_6$: 360.14, found: 360.15.

Dimethyl 4-(4-fluorophenyl)-2,6-dimethyl-1,4-dihydropyridine-3,5-dicarboxylate (5): white solid; ^{1}H NMR (400 MHz, $CDCl_3$): δ 7.24 (d, *J*=8.2 Hz, 2H, 2CH_{Ar}), 6.80 (d, *J*= 8.2 Hz, 2H, 2CH_{Ar}), 5.72 (bs, 1H, NH), 4.86 (s, 1H, CH), 3.44 (s, 6H, 2CH_3), 2.31 (s, 6H, 2CH_3); ^{13}C NMR (400 MHz, $CDCl_3$): δ 168.01, 145.08, 144.42, 129.11, 128.94, 126.38, 103.99, 51.56, 38.44, 19.67; LCMS (ESI) $[M+H]^+$ calcd for $C_{17}H_{18}NO_4F$: 320.13, found: 320.13

PRECURSOR INTERMEDIATE CHARACTERISATION

LC-HRMS analysis of precursor reaction mixture for nifedipine, containing 2-nitrobenzaldehyde (5 mmol) and methylacetoacetate (10 mmol) in DMF was carried out. Data indicated the presence of possible *E* and *Z* isomers of methyl-2-(2-nitrobenzylidene)-3-oxobutanoate (Figure 1, peak 1 and 2) and dimethyl 2,4-diacetyl-3-(2-nitrophenyl)pentanedioate intermediates (Figure 1, peak 3):

LCMS (ESI) $[M+H]^+$ calcd for $C_{12}H_{11}NO_5$: 250.0715, found: 250.0725; $[M+Na]^+$ calcd for $C_{12}H_{11}NO_5$: 272.0534, found: 272.0543.

$[M+H]^+$ calcd for $C_{17}H_{19}NO_8$: 366.1188, found: 366.1188; $[M+Na]^+$ calcd for $C_{17}H_{19}NO_8$: 388.1008, found: 388.1009.

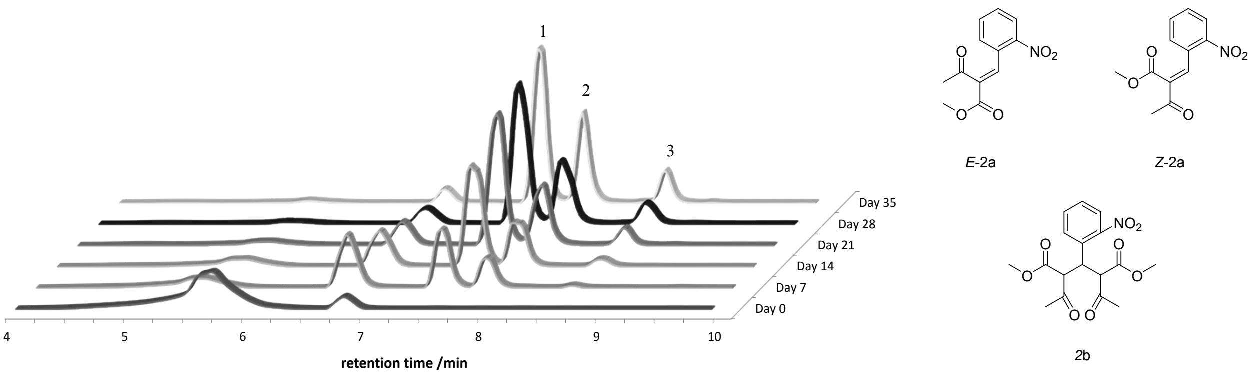

Figure 1. UV chromatograms of the nifedipine precursor mixture sampled over a 35-day time period. Peaks 1 and 2: isomers of M = 249.0637 (*E*-2a and/or *Z*-2a); peak 3: M= 365.1110 (2b)

For isolation of intermediates, semi-preparative HPLC was performed on an Agilent Technologies 1200 Series HPLC with GinaStar acquisition software using an Agilent Eclipse XDB-C_{18} column (5 µm, 9.4 × 250 mm); flow rate 3 mL/min; mobile phase: solvent A: H_2O + 0.1% TFA; solvent B: MeOH + 0.1% TFA; time (min): %B 0:5, 60:60, 70:60, 90:100, 100:100, 120:5. Fractions were collected manually and analysed by atmospheric pressure chemical ionisation mass spectrometry (APCI-MS) to confirm fraction identity, followed by lipholisation overnight. Samples were analysed on HPLC to assess purity. Any impure samples were re-purified again with semi-preparative HPLC using a slower gradient: time (min): %B 0:5, 5:30, 30:60, 35:60, 45:5, 120:5. Fractions were collected manually and

analysed by APCI-MS to confirm fraction identity, followed by lipholisation overnight. Samples were submitted for ^{1}H and ^{13}C NMR analysis. The identity of intermediate species *E*-2a and *Z*-2a was confirmed by ^{1}H and ^{13}C NMR. Although fractions containing 2b were also isolated, as evidenced by mass spectral analysis, NMR analysis showed that this intermediate decomposed in solution.

^{1}H and ^{13}C analysis

methyl-(*Z*)-2-(2-nitrobenzylidene)-3-oxobutanoate (*Z*-2a): ^{1}H NMR (400 MHz, CD_3CN): δ 8.23 (dd, *J* = 8.2, 1.1 Hz, 1H, CH_{Ar}), 8.07 (s, 1H, CH), 7.78 (td, *J* = 7.5, 1.1 Hz, 1H, CH_{Ar}), 7.68 (td, *J* = 8.2, 1.6 Hz, 1H, CH_{Ar}), 7.47(d, *J* = 7.7 Hz, 1H, CH_{Ar}), 3.60 (s, 3H, CH_3), 2.46 (s, 3H, CH_3); ^{13}C NMR (400 MHz, CD_3CN): δ 195.72, 166.93, 147.86, 141.20, 136.96, 134.90, 131.19, 130.82, 130.40, 125.60, 52.43, 26.79

methyl-(*E*)-2-(2-nitrobenzylidene)-3-oxobutanoate (*E*-2a): ^{1}H NMR (400 MHz, CD_3CN): δ 8.22 (dd, *J* = 8.2, 1.2 Hz, 1H, CH_{Ar}), 8.04 (s, 1H, CH), 7.74 (td, *J* = 7.5, 0.9 Hz, 1H, CH_{Ar}), 7.65 (td, *J* = 7.9, 1.7 Hz, 1H, CH_{Ar}), 7.38 (d, *J* = 7.6 Hz, 1H, CH_{Ar}), 3.87 (s, 3H, CH_3), 2.24 (s, 3H, CH_3); ^{13}C NMR (400 MHz, CD_3CN): δ 201.12, 165.00, 147.96, 140.52, 136.41, 134.76, 131.20, 130.99, 130.74, 125.57, 52.90, 31.05

Based on ^{1}H NMR integration, ***E*-2a** contains a small amount of impurities, <10%.

Stereoisomers ***Z*-2a** and ***E*-2a** were distinguished via 2D ^{1}H-^{1}H NOESY NMR: for ***Z*-2a** a cross-peak was observed between the ketone methyl group (δ 2.46) and the alkene proton (δ 8.07) (Figure 2).

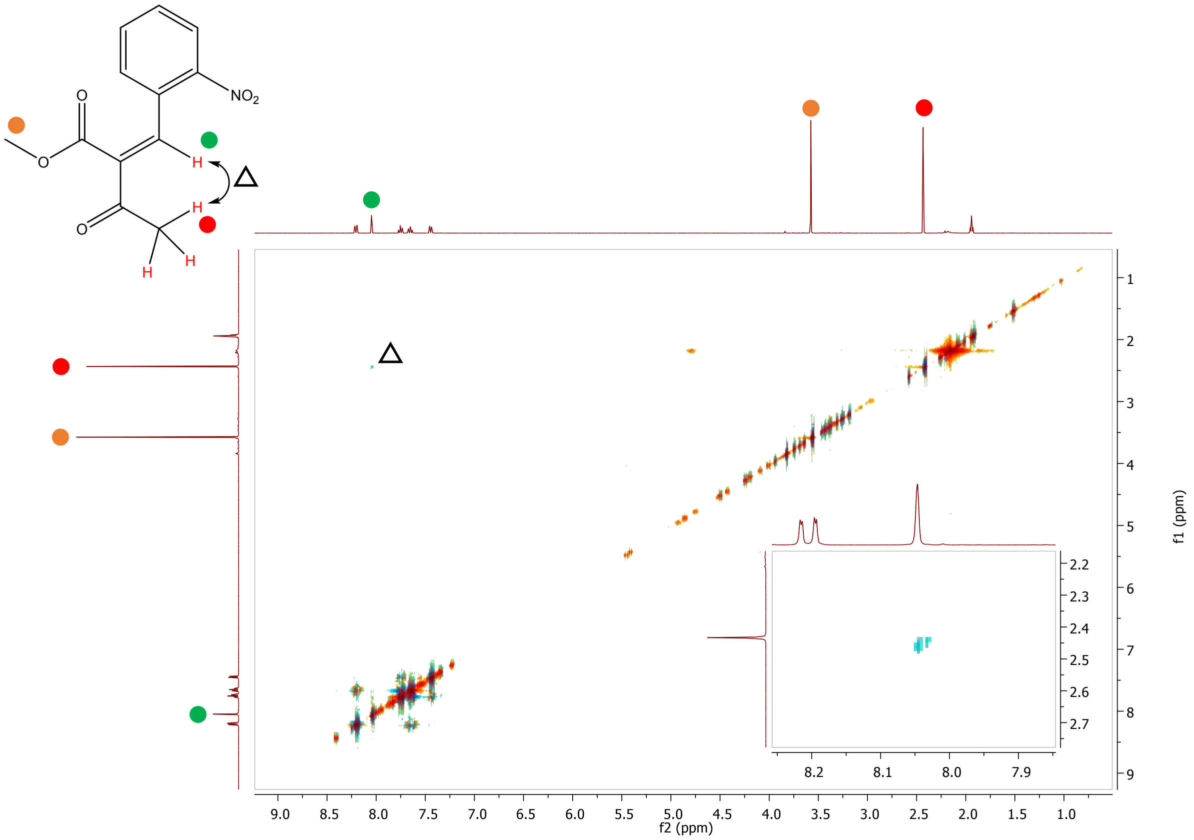

Figure 2. 2D ^{1}H-^{1}H NOESY NMR for methyl-(*Z*)-2-(2-nitrobenzylidene)-3-oxobutanoate **(*Z*-2a)**. Inset shows zoom of cross-peak at δ 8.07 (alkene proton) and δ 2.46 (ketone methyl group), denoted by △

RADIOLABELLING METHODS

Production of aqueous [^{13}N]NH_3

Aqueous [^{13}N]NH_3 was produced on a CTI RDS 112 biomedical cyclotron via the $^{16}O(p,\alpha)^{13}N$ nuclear reaction.[4] The target contained 8 mL H_2O with 5 mM ethanol and was irradiated with 11.2 MeV protons at a beam current of 30 µA for 20 min. The irradiated solution was pumped from the cyclotron through narrow bore PEEK tubing to the radiochemistry laboratory where it was passed through an IC-OH cartridge (Maxi-Clean™, Grace Davison Discovery Sciences) conditioned with water (5 mL), to remove impurities. The molar activity of [^{13}N]NH_3 was calculated to be 2.64 ± 0.12 GBq µmol^{-1}.

Radiolabelling optimisation

Aqueous [^{13}N]NH_3 (8 mL) from the cyclotron was concentrated into a smaller volume (1 mL) by loading on to a weak cation exchange Sep-Pak (Accell Plus CM Light 130 mg, Waters), rinsing with water (5 mL), and eluting with saline (0.9%, 1 mL). A stock precursor mixture was prepared: substituted benzaldehyde derivative (5 mmol) and methylacetoacetate (10 mmol) were combined in DMF (10 mL). From this stock solution, a 200 µL aliquot containing benzaldehyde derivative (0.1 mmol) and methyl acetoacetate (0.2 mmol) was used for each radiosynthesis. The aliquot was combined with an aqueous solution of NaOH (1 M, 5 µL) and [^{13}N]NH_3 (100 µL, 150 MBq). The mixture was heated in a microwave synthesiser at different temperatures and reaction time. A sample of the crude reaction mixture (20 µL) was analysed via radio-HPLC. Radiochemical yields are detailed in the main manuscript (Table 1, main manuscript).

High radioactivity preparation of ^{13}N-labelled 1,4-DHPs

Aqueous [^{13}N]NH_3 (1.5 GBq) was trapped on a cation exchange Accell Plus CM Light Sep-Pak cartridge (130 mg, conditioned with water followed by air). The [^{13}N]NH_3 was eluted with sodium chloride solution (250 µL, 1 M) into a microwave vial containing precursor mixture consisting of methyl acetoacetate (0.2 mmol) and benzaldehyde derivative (0.1 mmol) in DMF (200 µL), and aqueous NaOH (1 M, 5 µL). The mixture was heated in the microwave at 100 ^{o}C for 5 min. The mixture was then cooled and diluted with methanol (500 µL), before HPLC purification. The radioactive [^{13}N]1,4-DHP

fraction eluted at ~ 8 min and was collected in approximately 3 mL of HPLC mobile phase containing MeOH, water and TFA. The radioactive [^{13}N]1,4-DHP HPLC fraction was diluted with water (3 mL) and loaded on to a C_{18} Plus Light Sep-Pak (130 mg, conditioned with methanol, then water, and dried with air). The final product was eluted from the cartridge with ethanol (300 µL), and saline (2.7 mL, 0.9%) was slowly added to the solution for final reconstitution. A sample of the final tracer solution was analysed using radio-HPLC (Figure 3). The molar activity of the final product was calculated for [^{13}N]nifedipine using HPLC: the area under the curve of the UV signal of known concentrations of non-radioactive nifedipine standard was used to derive a standard curve. This was used to calculate the unknown concentration of nifedipine in the radioactive [^{13}N]nifedipine sample by integrating the area under the curve of the signal in the UV.

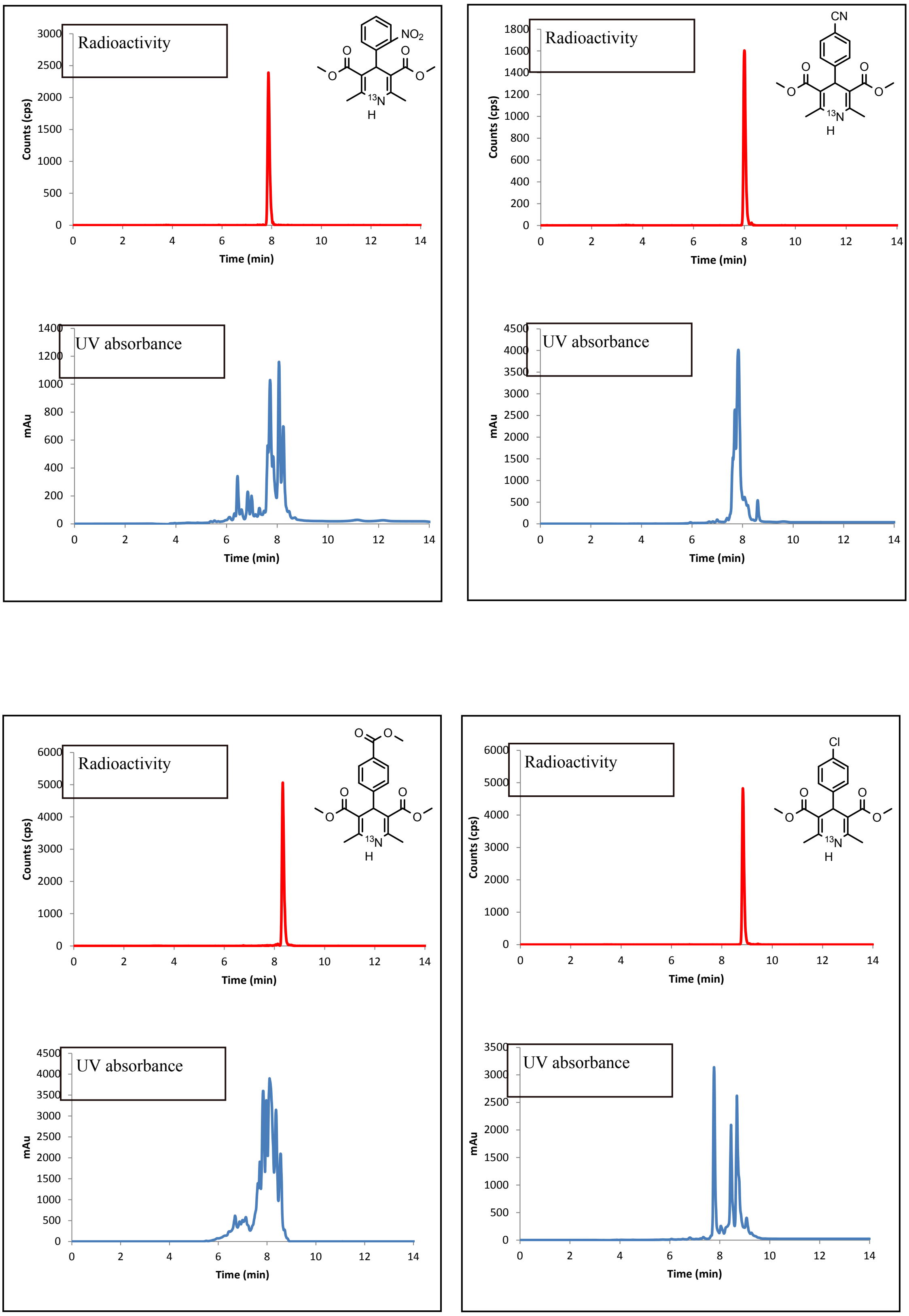

Figure 3. HPLC radio-chromatogram of the ^{13}N-labelled 1,4-DHPs, after semi-preparative purification. Red: radioactivity (counts per second); blue: UV absorption (254 nm). Whilst the radiochromatograms show high radiochemical purity for each ^{13}N-labelled derivative (> 99 %), UV traces for all radiolabelled compounds show the presence of multiple non-radioactive species.

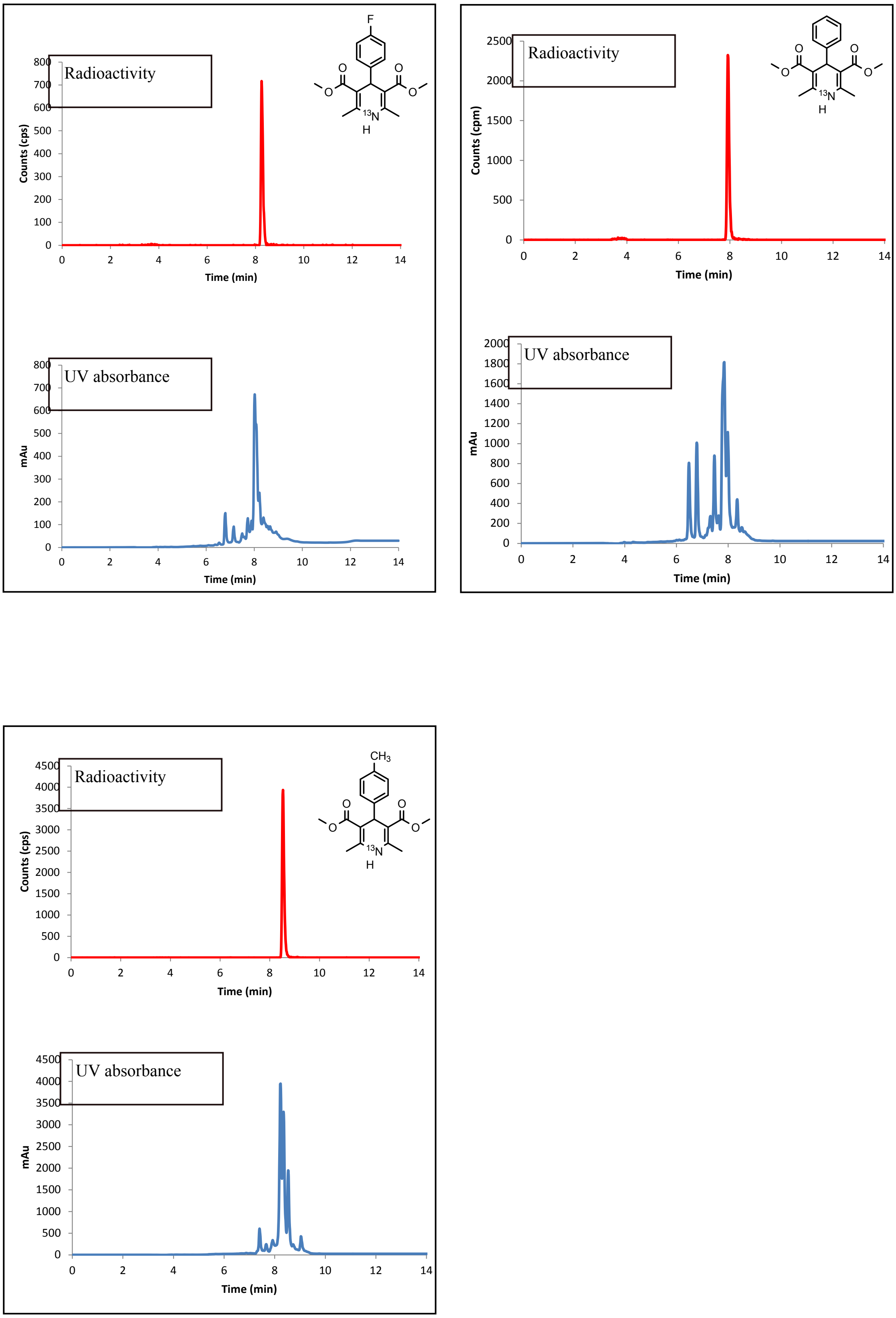

Figure 3 (cont.). HPLC radio-chromatogram of the ^{13}N-labelled 1,4-DHPs, after semi-preparative purification. Red: radioactivity (counts per second); blue: UV absorption (254 nm). Whilst the radiochromatograms show high radiochemical purity for each ^{13}N-labelled derivative (> 99 %), UV traces for all radiolabelled compounds show the presence of multiple non-radioactive species.

MICROPET/CT IMAGING AND BIODISTRIBUTION

All animal experiments in this study were performed under Home Office licence number PPL70/7019; this licence was approved by KCL AWERB (Animal Welfare and Ethical Review Board) prior to submission and granting of authorisation by the UK government Home Office. All experimental designs were in line and performed in accordance with the Animals (Scientific Procedures) Act, 1986, 2012 amendment.

PET imaging and biodistribution of [^{13}N]nifedipine was performed on female Wistar rats. Rats were kept in standard local animal housing conditions housing conditions and fed *ad libitum* with regular animal feed.

<u>MicroPET/CT Imaging</u>

Imaging experiments were performed using a nanoScan® PET/CT (123 mm transaxial FOV, 0.98 mm spatial resolution) (Mediso Medical Imaging Systems, Budapest, Hungary). A rat was anaesthetised under 1 L/min O_2 flow rate with 3% isoflurane (Vet Tech Solution Ltd.). [^{13}N]Nifedipine (~4 MBq, 46 µmol in 800 µL 0.9% saline, 10% EtOH) was injected via tail vein using a catheter while the animal was on the scan bed. Isofluorane inhalation (1.5-3%) was achieved using a facemask for the duration of the scan (Equipment veterinaire Minerve). Dynamic upper-body PET imaging (energy window 400-600 keV; coincidence relation 1:3) was acquired for 1 h post-administration of the tracer, followed by a 5-7 min CT scan (180 projections, pitch 1). Respiration rate was monitored for the duration of the imaging process. At the end of the imaging experiment, the rat was culled using terminal anaesthesia (1 L/min O_2 flow with 5% isofluorane) followed by cervical dislocation. All PET/CT images acquired were processed and analysed using Vivoquant 1.21 (Invicro Imaging Services and Software), which enables the overlay of PET and CT images. For quantification of PET signal, regions of interest (ROI's) were drawn manually within the heart, right common carotid artery (blood), liver and brain. A single ROI that is 4-pixels in size was drawn for each organ. The heart ROI was drawn close to the periphery of the ventricle in an attempt to capture signal within heart muscle, avoiding blood pool signal from the ventricular cavity.

Ex vivo biodistribution

A rat was anaesthetised under 1 L/min O_2 flow rate with 3 % isofluorane. [^{13}N]Nifedipine (~ 4.5 MBq in 600 µL of 0.9 % saline, 10% EtOH) was administered via tail vein. Anaesthesia was maintained by isofluorane inhalation (1.5-3 %) using a face mask for the duration of the experiment. At 25 min post-injection, the rat was culled using terminal anaesthesia (1 L/min O_2 flow with 5% isofluorane) followed by cervical dislocation. Tissues of interest were extracted and washed in PBS buffer to remove residual blood. The tissue was transferred to weighed tubes for weighing and measurement in the gamma counter (LKB Wallac 1282 Compugamma Universal). The injected dose was calculated by measuring the weight of the syringe before and after injection. Accumulation of radioactivity in each organ was expressed as percentage of injected dose (% ID, Figure 4) and percentage of injected dose per gram (% ID/g, Figure 5).

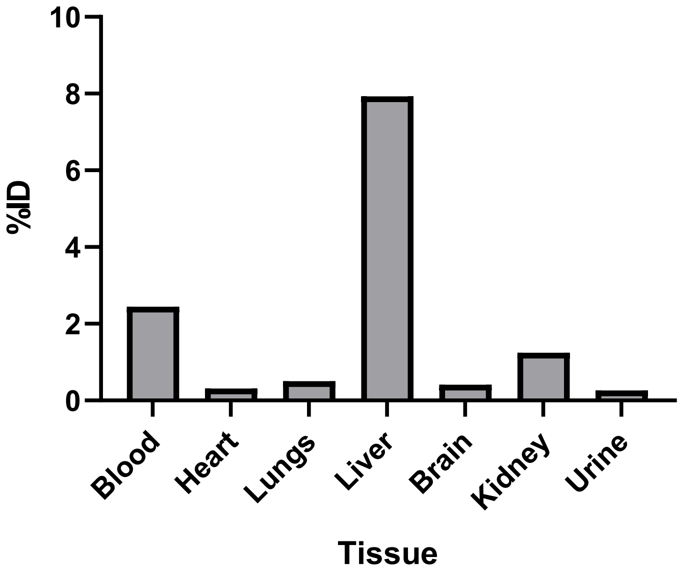

Figure 4. %ID of [^{13}N]nifedipine in normal rat at 30 min post-injection (n=1)

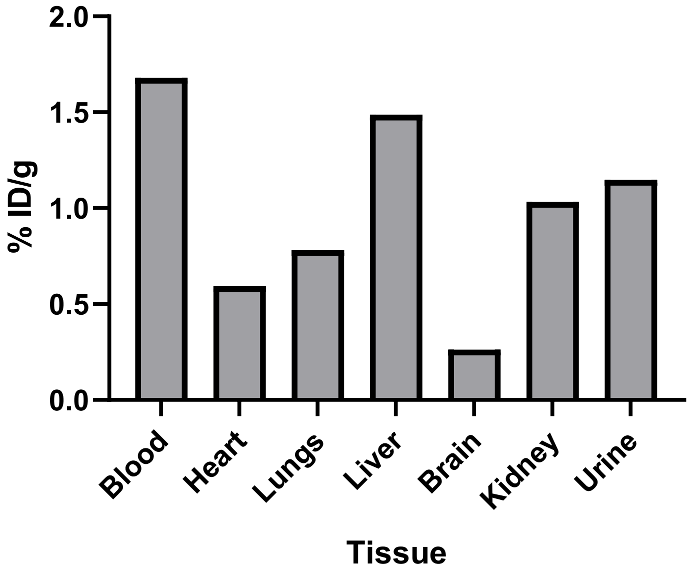

Figure 5. %ID/g of [^{13}N]nifedipine in normal rat at 30 min post-injection (n=1)

REFERENCES

1. M. F. Gordeev, D. V. Patel and E. M. Gordon, *J Org Chem,* 1996, **61**, 924-928
2. J. Legeay, J. J. V. Eynde and J. P. Bazureau, *Tetrahedron*, 2005, **61**, 12386-12397
3. A. G. Sathicq, G. P. Romanelli, A. Ponzinibbio, G. T. Baronetti and H. J. Thomas, *Lett Org Chem*, 2010, **7**, 511-518
4. B. Wieland, G. Bida, H. Padgett, G. Hendry, E. Zippi, G. Kabalka, J. L. Morelle, R. Verbruggen and M. Ghyoot, *Int J Rad Appl Instrum A*, 1991, **42**, 1095-1098.

ACKNOWLEDGEMENTS

The authors wish to acknowledge the expertise of Matthew Farleigh in preparation of Figure 2.
